# Inactivation of Interferon Regulatory Factor 1 Causes Susceptibility to Colitis-Associated Colorectal Cancer

**DOI:** 10.1038/s41598-019-55378-2

**Published:** 2019-12-11

**Authors:** Thiviya Jeyakumar, Nassima Fodil, Lauren Van Der Kraak, Charles Meunier, Romain Cayrol, Kevin McGregor, David Langlais, Celia M. T. Greenwood, Nicole Beauchemin, Philippe Gros

**Affiliations:** 1 Department of Biochemistry, McGill University, Montreal, QC Canada; 2 Department of Human Genetics, McGill University, Montreal, QC Canada; 3 Goodman Cancer Research Centre, McGill University, Montreal, QC Canada; 4 Department of Oncology, McGill University, Montreal, QC Canada; 5 Department of Medicine, McGill University, Montreal, QC Canada; 6 Department of Epidemiology, Biostatistics and Occupational Health, McGill University, Montreal, QC Canada; 7 Lady Davis Institute for Medical Research, Jewish General Hospital, Montreal, QC Canada; 8 Département de Pathologie et de Biologie Cellulaire, Université de Montréal, Montreal, QC Canada

**Keywords:** Colon cancer, Cancer genetics, Inflammatory bowel disease, Chronic inflammation

## Abstract

The mechanisms linking chronic inflammation of the gut (IBD) and increased colorectal cancer susceptibility are poorly understood. IBD risk is influenced by genetic factors, including the *IBD5* locus (human 5q31), that harbors the *IRF1* gene. A cause-to-effect relationship between chronic inflammation and colorectal cancer, and a possible role of *IRF1* were studied in *Irf1*^*-/-*^ mice in a model of colitis-associated colorectal cancer (CA-CRC) induced by azoxymethane and dextran sulfate. Loss of *Irf1* causes hyper-susceptibility to CA-CRC, with early onset and increased number of tumors leading to rapid lethality. Transcript profiling (RNA-seq) and immunostaining of colons shows heightened inflammation and enhanced enterocyte proliferation in *Irf1*^*−/−*^ mutants, prior to appearance of tumors. Considerable infiltration of leukocytes is seen in *Irf1*^*−/−*^ colons at this early stage, and is composed primarily of proinflammatory Gr1^+^ Cd11b^+^ myeloid cells and other granulocytes, as well as CD4^+^ lymphoid cells. Differential susceptibility to CA-CRC of *Irf1*^*−/−*^ vs. B6 controls is fully transferable through hematopoietic cells as observed in bone marrow chimera studies. Transcript signatures seen in *Irf1*^*−/−*^ mice in response to AOM/DSS are enriched in clinical specimens from patients with IBD and with colorectal cancer. In addition, *IRF1* expression in the colon is significantly decreased in late stage colorectal cancer (stages 3, 4) and is associated with poorer prognosis. This suggests that partial or complete loss of *IRF1* expression alters the type, number, and function of immune cells *in situ* during chronic inflammation, possibly via the creation of a tumor-promoting environment.

## Introduction

Colorectal cancer was the third most commonly diagnosed cancer worldwide in 2018^1^. The etiology of colorectal cancer involves both a strong genetic contribution and complex environmental and socioeconomic determinants including diet, lifestyle, and smoking^2,3^. A positive risk factor for colorectal cancer is a prior history of chronic intestinal inflammation. Indeed, patients with inflammatory bowel disease (IBD) are at greater risk of developing colorectal cancer than the general population^4^. IBD represents intestinal disorders that comprise Crohn’s disease (CD) and ulcerative colitis (UC) resulting in lifelong chronic inflammation of different segments of the intestine^5^. Colitis-associated colorectal cancer (CA-CRC) is the most severe consequence of IBD.

Population studies have demonstrated that in humans, IBD is under complex genetic control with >225 risk loci mapped in large cohort association studies (GWAS)^6–8^. However, the specific contributions of these loci, individually or as a group, to CA-CRC risk remain unknown. Such genetic loci may encode proteins of significant value as potential drug targets, and as predictive markers to identify IBD patient subsets with increased risk of developing CA-CRC. For instance, the *IBD5* risk locus (5q31-q33) was first associated to CD by genome scan in 158 Canadian sib-pair families with segregating disease^9^. The *IBD5* association was replicated and refined by GWAS to a region that maps to a segment of human Chromosome 5 (5q31.1) containing ~20 genes in linkage disequilibrium with a number of attractive positional candidates, including the transcription factor Interferon regulatory factor 1 (*IRF1*)^7^. Although multiple genes at the *IBD5* locus were studied for their putative causality under the associated haplotype^10^, to date, none have been functionally validated.

IRF1 is a transcription factor that plays a key role in the development and function of myeloid and lymphoid cells. IRF1 is expressed constitutively at low levels in many cell types and is induced in response to various stimuli, including viral and bacterial infections, and cytokines such as IFNγ and TNFα^11^. Upon induction, IRF1 stimulates transcription of IFN-inducible genes involved in innate and acquired immunity. *Irf1*-deficient mice display a severe immunodeficiency, with alterations in myeloid cell maturation, CD8^+^ T cell numbers, and impaired NK cell cytolytic activity^12–15^. *Irf1*^*−/−*^ mice show active colitis in response to the irritant dextran sodium sulfate (DSS)^16,17^, and human *IRF1* mRNA is increased in lamina propria mononuclear cells from CD patients^18^, thus suggesting that IRF1 may contribute to inflammation in IBD, and possibly to progression to CA-CRC.

We have tested a possible role of IRF1 in CA-CRC, using a combination of studies in mice and humans. We found that *Irf1*^*−/−*^ mice display increased tumorigenesis in response to azoxymethane and dextran sodium sulfate (AOM/DSS)-induced CA-CRC. Combination of immune cell profiling and comparative transcriptome analysis of the colon by RNA-seq show that the increased susceptibility phenotype of *Irf1*^*−/−*^ mutants is linked to increased inflammation and immune cell infiltration in the colon of *Irf1*^*−/−*^ mice, and is transferable by hematopoietic cells in bone marrow chimera experiments. Genes dysregulated in *Irf1*-deficient mouse colon are enriched in gene set hallmarks of active UC and IBD. In addition, analysis of The Cancer Genome Atlas (TCGA) database for *IRF1* expression in human colorectal cancer patients revealed a statistically significant reduction of *IRF1* in colorectal cancer patient tumors of Stage 1, 3, and 4. Together, these results suggest a critical role for IRF1 in regulating the type and intensity of inflammatory response in the colon, contributing to establishment of permissive conditions for progression of IBD to CA-CRC.

## Materials and Methods

### Ethic statement

All animal experimentation was performed in accordance with the guidelines and regulations of the Canadian Council on Animal Care (CCAC) and were approved by the McGill University Animal Care and Ethics Committee.

### Mouse models of CA-CRC, CRC and IBD

For the CA-CRC model^19^, 8–10 week-old C57BL/6J, A/J, or *Irf1*^*−/−*^ mice (The Jackson Laboratory) were injected ip with azoxymethane (AOM; 7 mg/kg) followed by three 4-day cycles of 2% (w/v) DSS in the drinking water, with each cycle 17 days apart and euthanized 6 weeks later. In some experiments, mice were injected with AOM (7 mg/kg) once, followed by two 4-days cycles of 2% (w/v) DSS and sacrificed 3 weeks later. The model of AOM-induced colorectal cancer was as previously described^20^. For the chronic colitis model, mice were treated with three 4-days cycles of 2% (w/v) DSS and sacrificed after week 8.

### Histological analysis

Formalin-fixed, paraffin-embedded tissue sections were stained with hematoxylin/eosin, were scored for markers of inflammation as previously described^21^.

### Immunoprofiling by flow cytometry

Colons were cut into ~1 cm^2^ pieces, washed in 1X Hank’s Balanced Salt Solution (HBSS) containing 2% heat-inactivated fetal bovine serum (FBS), disrupted by vortexing, further treated with collagenase IV (20 mg/ml) and DNase I (10 mg/mL) in RPMI medium and passed through a 70 μM cell strainer to collect lamina propria cells. Cells were treated with blocking antibodies to CD16/32 (1:100), and stained for viability using Zombie Viability Dye V500 (1:400). Surface staining was done on ice for 20 min using anti-CD45 APC-eFluor780 (1:400, clone 30F11, eBioscience), anti-CD11b APC (1:400, clone M1/70, InVitrogen), anti-Gr1 PerCp-Cy5.5 (1:300, clone RB6-8C5, Biolegend), anti-Siglec F PE (1:400, clone E50-2440, BD), anti-CD3 FITC (1:200, clone 145-2C11, Biolegend), anti-CD49b FITC (1:200, clone DX5, eBioscience), anti-B220 FITC (1:300, clone RA3-6B2, eBioscience), anti-CD4 PerCp-Cy5.5 (1:400, clone RM4-5, eBioscience), and anti-CD8a AlexaFluor 700 (1:200, clone 53–6.7, Biolegend),. Intestinal epithelial cells were collected as described in^22^. Cells were stained with anti-EpCAM PE-Cy7 (1:400, clone G8.8, eBioscience) and anti-FITC CD44 (1:200, clone IM7, eBioscience). Cells were analyzed on the Fortessa (BD Biosciences) and data analysis was performed on FlowJo (Tree Star).

### RNA-sequencing

Mice received one injection of AOM (D1) followed by one 4-day cycle of DSS (2%; D8-D11) and were sacrificed at D26. Distal colons were collected and flash-frozen in liquid nitrogen. RNA was isolated from homogenized tissue with TRIzol reagent (Life Technologies) and purified using an RNeasy Mini Kit (Qiagen). RNA integrity was ascertained using a Bioanalyzer Total RNA Pico assay (Agilent). rRNA-depleted libraries were prepared using Kapa-stranded RNA-seq kit (Kapa Biosystems), sequenced as previously described^23^ and analyzed using publicly available bioinformatics work tools and software packages^24–27^. Differential gene analysis was evaluated as described^28^ where genes with <5 counts per million for at least 3 samples were filtered out. Genes were considered significantly differentially expressed if fold change was ≥1.5 or ≤−1.5 and FDR <0.01. Gene ontology (GO) enrichment analysis was performed using DAVID bioinformatics resources v6.8^29^ and gene set enrichment analysis (GSEA) was analyzed using the GSEA software^30^. Gene sets from published data can be accessed at GSE4183. Relative abundance of certain transcripts was verified by quantitative PCR (qPCR), as described^23^. Primers for genes tested are listed in Supplementary Table 1. mRNA levels were quantified using the comparative CT method^31^ and were normalized to expression of *Hprt*.

### Immunofluorescence analysis

Colons were collected from untreated and D26 post-AOM/DSS-treated B6 and *Irf1*^*−/−*^ mice, fixed in 4% paraformaldehyde for 6 h at 4 °C, cryo-protected in 20% sucrose, embedded and frozen in optimal cutting temperature compound. Primary antibodies used were anti-CD11b (1:100, clone M1/70, eBioscience), and anti-FITC Ki67 (1:100, clone SolA15, InVitrogen). Goat anti-rat secondary antibodies conjugated with DyLight 488 were used at a 1:1,000 dilution. Immunostained sections were counterstained with DAPI (1:1,000) to visualize nuclei. For quantification of proliferation, at least 5 fields were selected randomly, and all crypts were counted under 10X magnification. Crypts were considered “hyperproliferative” if Ki67^+^ cells extended from the base to at least 3/4 of the height of the crypts; the percentage of hyperproliferative crypts/normal crypts was calculated from total counts. All images were acquired on a Zeiss Axiovert 200 M Automated Inverted microscope.

### Bone marrow chimeras

CD45.1 and *Irf1*^*−/−*^ mice, 8 weeks-old, were irradiated with 450 rad twice within a 3 hour interval on an X-Ray RS-2000 Biological Irradiator. After 3 hours, mice were injected intravenously with 5 × 10^6^ red blood cell-depleted bone marrow from female donors. Mice received 50 mg/mL enrofloxacin (Baytril) in their drinking water 3 days prior to irradiation and for 3 weeks post-irradiation. Bone marrow reconstitution was verified at 8 weeks post-transplantation by flow cytometry. At 9 weeks post-engraftment, mice were subjected to AOM-DSS treatment as described above.

### Gene expression microarray analysis

Raw microarray data generated from colon biopsies of patients with either active UC (UC_act) or those in remission (UC_rem), together with control non-inflamed tissue of UC patients and tissue from non-UC patients were obtained from Gene Expression Omnibus repository (GSE38713)^32^. Data are GC-RMA pre-processed log2 fluorescence intensities^33^ from Affymetrix Human Genome U133 Plus 2.0 Arrays. Intensities were quantile-normalized to reduce the inter-sample variations. Probesets with no or very low signals were filtered out, thus only probesets with a log2 fluorescence intensity of >5 for at least 3 samples were retained for subsequent analyses. Differential gene expression analysis was performed by comparing UC_act or UC_rem groups with the control group. Two statistical tests were applied to determine significance of differential expression: LIMMA and Bonferroni-corrected Student’s T-test; genes with a fold change ≥2 or ≤0.5 and with an adjusted p value ≤ 0.05 in both tests were considered statistically dysregulated.

### TCGA analysis of human colon tumors

Data were analyzed from the January 2016 version of the TCGA colon and rectal adenocarcinoma (COADREAD) dataset. When colon and rectal carcinomas were sequenced on both Illumina HiSeq & GA platforms, only the HiSeq results were used. Zero reads were replaced with a random number based on the mean and standard deviation of very low read numbers (with the number of reads <1). Two normalization methods were used, quantile normalization of all samples, and a Z-transformation of batches with at least 10 samples. For the Z-transformation, the overall mean of gene expression in each batch was Z-transformed to have a mean 0 and a standard deviation of 1. This gauged if a gene was expressed at a high or low level within this specific batch, and corrected for batch-specific effects. The batch-specific Z-filtered and quantile scores were used in all further survival calculations^34^.

### Statistical analyses

Except when otherwise indicated, data were analyzed with GraphPad Prism 5 software (GraphPad Software), and are presented as mean ± SD. The significance of weight loss measurements was evaluated by two-way ANOVA, followed by Bonferroni’s multiple-comparison test. Mann-Whitney test was used for analysis of histopathological scoring. A one-way ANOVA followed by Bonferroni’s multiple-comparison test was employed to compare proportions of intestinal proliferation. Fisher’s exact test was used to compare gene expression profiles of AOM/DSS-treated *Irf1*^*−/−*^ and B6 mice with human UC patients. Unpaired, two-tailed Student’s t-tests were used for all other determinations of significant differences between two groups.

### Accession codes

GEO: RNA-seq data: GSE116374.

## Results

### *Irf1*^*−/−*^ mice are highly susceptible to colitis-associated colorectal cancer

**Figure 1:**
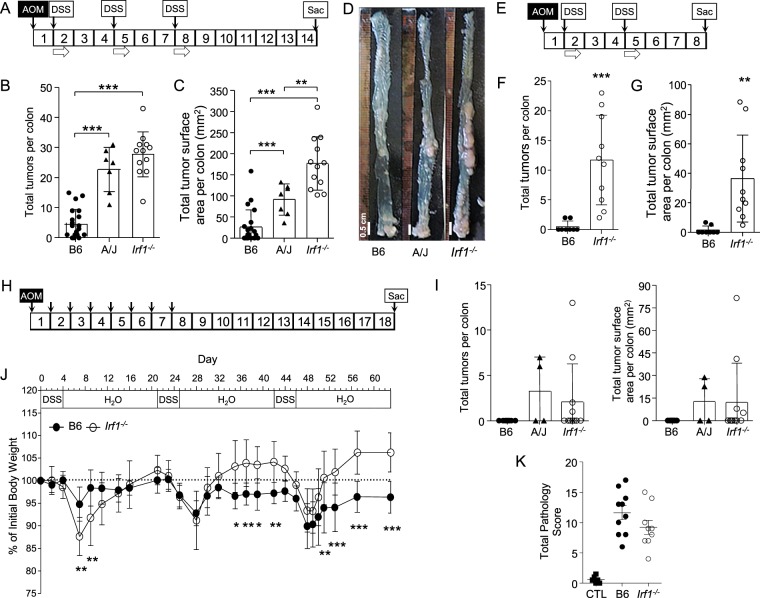
*Irf1*^*−/−*^ mice show susceptibility to colitis-associated colorectal cancer (CA-CRC) induced by combined treatment with azoxymethane and dextran sulfate (AOM/DSS). (**A**) Experimental protocol: Mice were injected ip with AOM (7 mg/kg) once followed by three cycles of 2% (w/v) DSS in drinking water (4 days each) and sacrificed 6 weeks after the final DSS treatment. CA-CRC susceptibility in treated control resistant C57BL/6J (B6, n = 21), and susceptible A/J mice (A/J, n = 7) along with *Irf1*^*−/−*^ mutants (*Irf1*^*−/−*^, n = 12) animals was assessed by determining the number (**B**) and total surface areas of tumors (**C**) on freshly dissected colons with representative photographs shown (**D**). (**E**) Experimental protocol: Mice were injected ip with AOM (7 mg/kg) once followed by two cycles of 2% (w/v) DSS in drinking water (4 days each) and sacrificed 3 weeks after the final DSS treatment. CA-CRC susceptibility in treated B6 (n = 8) and *Irf1*^*−/−*^ mice (n = 10) as assessed by determining the number (**F**) and total surface areas of neoplastic lesions (**G**) on freshly dissected colons. Statistical significance of inter-group differences was assessed using unpaired Student’s t-tests (*<0.05, **<0.01, ***<0.001). (**H**) Experimental protocol: Mice (control resistant B6, n = 11; control susceptible A/J, n = 4, *Irf1*^*−/−*^ mutant, n = 11) were injected ip with AOM (7 mg/kg) once a week for 8 weeks and sacrificed 11 weeks later. Susceptibility to AOM-induced CRC was measured as tumor multiplicity and tumor surface area (**I**). Colitis was induced by chronic exposure to DSS (J): Mice (control B6, n = 10; *Irf1*^*−/−*^ mutant, n = 9) were treated with three cycles of 2% (w/v) DSS in drinking water (4 days each) and were sacrificed 3 weeks after the final DSS treatment. Body weight was monitored and changes are expressed as percentage of initial weight prior to treatment (**J**). A two-way ANOVA with Bonferroni post-hoc evaluations was used to determine significance (*<0.05, **<0.01, ***<0.001). Colitis-associated pathological changes were determined by histological evaluation of colons (**K**) of untreated control B6 mice (CTL, n = 6) and of treated B6 or *Irf1*^*−/−*^ mutants. A total pathological score (0–36) was determined, as described in Materials and Methods.

Considering the established role of IRF1 in regulation of inflammatory response, including its possible implication as a risk factor in IBD^7^ and colorectal cancer^35^, we tested the possible role of Irf1 in CA-CRC. For this, we compared the response of resistant C57BL/6J (B6) control and *Irf1*^*−/−*^ mutant mice (on a B6 background) to AOM/DSS treatment (Fig. 1), with inbred strain A/J as a positive susceptible control^19^. Mice received a single dose of AOM followed by 3 treatments of 4 days with 2% DSS (Fig. 1A). While resistant B6 mice developed few tumors (0 < n < 15; Fig. 1B) with an average total neoplastic surface area of ~20 mm^2^ (Fig. 1C,D), A/J controls displayed a higher number of tumors (10 < n < 30; Fig. 1B) with a greater average total neoplastic surface area of ~90 mm^2^ (Fig. 1C,D)^19^. In contrast, *Irf1*^*−/−*^ mutants were highly susceptible to AOM/DSS, with a high number of tumors (12 < n < 42; Fig. 1B) and an average total neoplastic surface area of ~170 mm^2^ (Fig. 1C,D). We conducted a second experiment in which B6 and *Irf1*^*−/−*^ mutants received AOM and only two 2% DSS treatments, with mice sacrificed at the end of week 8. In contrast to the B6 control mice, *Irf1*^*−/−*^ mutants were still highly susceptible in this less aggressive protocol, with many tumors observed with an average total neoplastic surface area of ~38 mm^2^ (Fig. 1E–G). Histological examination of tumors showed well-formed adenomas, as previously described (Fig. S1A,B)^36^. These results show that loss of *Irf1* causes susceptibility to CA-CRC in mice.

The hypersensitivity of *Irf1*^*−/−*^ mutants to CA-CRC induced by AOM/DSS could result from increased sensitivity to AOM-induced tumorigenesis or, from altered inflammation following exposure to DSS, or the combination of both. Carcinogen-induced CRC caused by serial (n = 8) treatments with AOM, in absence of DSS treatment^20^ (Fig. 1H) was not impacted in *Irf1*^*−/−*^ mutants with respect to number and size of tumors (at end of week 18; Fig. 1I), in comparison with the resistant B6 and susceptible A/J mice. The effect of *Irf1* inactivation on response to intestinal inflammation and injury was then tested in mouse models of colitis induced by chronic exposure to DSS without AOM treatment. Controls and *Irf1*^*−/−*^ mutants were exposed to 3 × 4-day regimens of 2% DSS at weeks 0, 3, and 6 and body weight was monitored daily. Nine weeks post initiation, colitis was assessed by histological examination. B6 and *Irf1*^*−/−*^ mutants showed similar responses with respect to cyclical fluctuations of body weight following each round of DSS (Fig. 1J) and pathology scores (Figs. 1K, S1C). Although *Irf1*^*−/−*^ mice appeared to initially lose more weight than B6 following the first DSS cycle, they regained weight more rapidly and to a greater extent than B6 upon subsequent cycles (Fig. 1J). These results suggest that *Irf1*^*−/−*^ mutants are not more susceptible to chronic DSS treatment than control B6 mice. Together, these data indicate that hyper-susceptibility of *Irf1*^*−/−*^ mutants to CA-CRC is not linked to increased susceptibility to AOM-induced carcinogenesis *per se*. Likewise, *Irf1*^*−/−*^ mutants and control mice develop a similar pathology in response to chronic treatment with DSS. However, *Irf1*^*−/−*^ mice exhibit a unique susceptibility to the combination of both treatments.

### Irf1-dependent regulation of gene expression in the colon during CA-CRC

**Figure 2:**
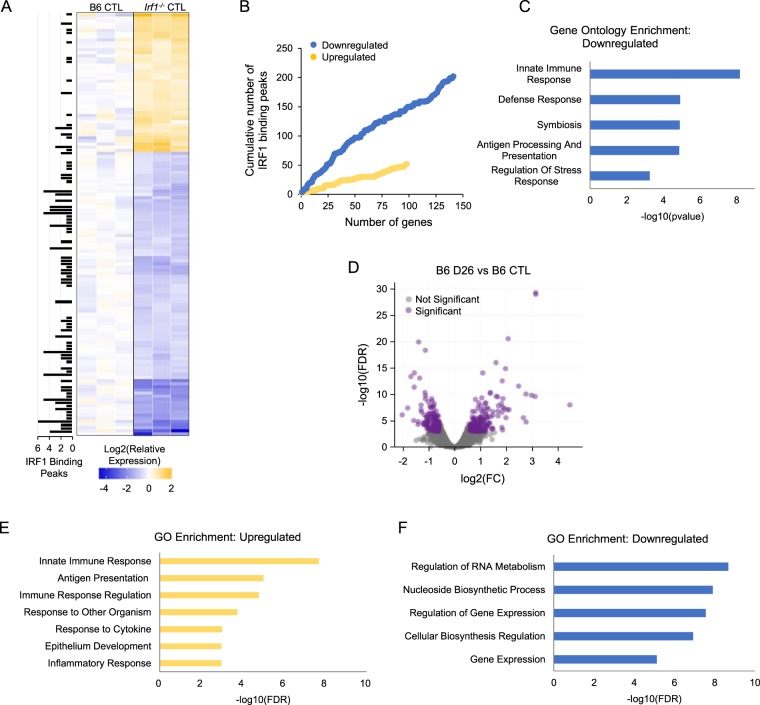
Transcriptional changes in the colon of *Irf1*^*−/−*^ mutants at steady state and B6 mice following treatment with AOM/DSS. RNA sequencing was conducted on total RNA from the colons of control B6 and *Irf1*^*−/−*^ mice; Differential gene analysis (statistically measured by fold change, FC, and false discovery rate, FDR) was performed as described in Materials and Methods. Significantly upregulated (FC ≥1.5, FDR ≤10^−2^, n = 44) and downregulated genes (FC ≤−1.5, FDR ≤10^−2^, n = 90) are displayed as a heat map (**A**), and total number of Irf1 binding peaks (determined by ChIP sequencing; Langlais et al., 2016) in the vicinity of each gene is shown in the heat map (**A**). Cumulative enrichment of Irf1 binding peaks near downregulated (blue) vs. upregulated (yellow) genes is shown (**B**). Gene Ontology (GO) analysis for enrichment of specific GO terms in the group of downregulated genes is shown (**C**). RNA sequencing was performed on total RNA from the colons of control and treated B6 (at day 26 post-treatment); Genes differentially expressed between control and treated B6 samples were identified, as described in (A). Significantly upregulated (FC ≥1.5, FDR ≤10^−2^, n = 261) and downregulated genes (FC ≤−1.5, FDR ≤10^−2^, n = 205) are displayed by volcano plot (**D**). Gene Ontology (GO) analysis for enrichment of specific GO terms in the group of upregulated (**E**) and downregulated (**F**) genes for the control B6 vs. treated B6 is shown.

RNA expression profiling was used to investigate the impact of *Irf1* inactivation on the colon transcriptome, at both steady state and following AOM/DSS treatment. To identify genotype-associated gene expression changes that might predispose to CA-CRC, RNA-seq was first performed at steady-state (D0) on colons from B6 controls and *Irf1*^*−/−*^ mutants. Differentially expressed genes in *Irf1*^*−/−*^ relative to B6 colons (Fig. 2A) were compared to Irf1 cistrome (genes bound by Irf1) and transcriptome (regulated by Irf1) gene lists which revealed a significant enrichment of Irf1 binding events at the regulatory sequences of down-regulated genes vs. up-regulated genes (Fig. 2B). These downregulated genes were enriched in GO terms for Innate Immune Response, Defense Response, Antigen Processing and Presentation (Fig. 2C). These findings infer that absence of Irf1 alters the colonic microenvironment linked in part to transcriptional programs driven by Irf1 in immune cells. This possibly reflects changes in the numbers and/or function of these cells in the *Irf1*^*−/−*^ intestinal mucosa.

We then evaluated transcriptional changes in the colons of control B6 mice in response to AOM/DSS treatment (Fig. 2D). The top GO terms associated with the upregulated genes were related to immune responses, antigen presentation and epithelium development (Fig. 2E), whereas downregulated genes were primarily those involved in transcription and translation (Fig. 2F). These results suggest strong activation of immune responses and tissue repair in response to AOM/DSS-induced in B6 mice. A similar analysis of *Irf1*^*−/−*^ mutant response to AOM/DSS (untreated vs. treated *Irf1*^*−/−*^ mutants, D26) was conducted (Fig. S2A,B). GO terms enriched in upregulated genes included Inflammatory Response and Leukocyte Activation (Fig. S2C) and the GO term Muscle Contraction was enriched in downregulated genes (Fig. S2D). This response is indicative of active inflammation and immune cell infiltration in *Irf1*^*−/−*^ colons in response to AOM/DSS.

**Figure 3:**
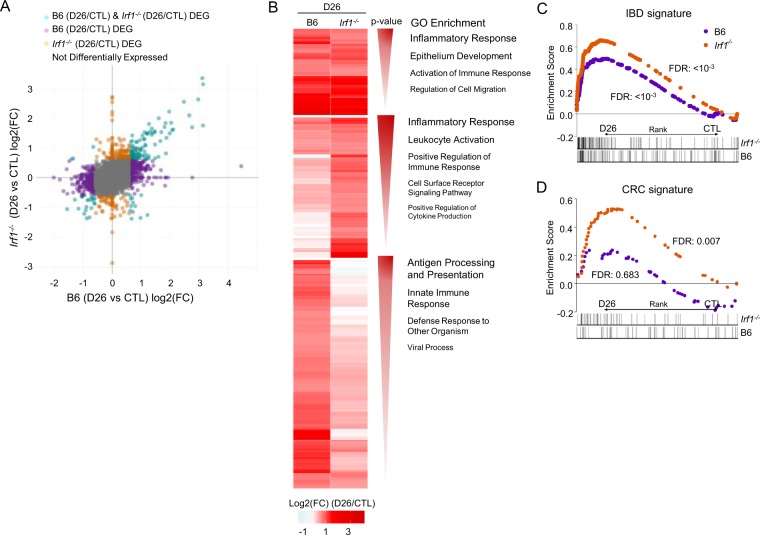
Differential transcriptional response of *Irf1*^*−/−*^ mutants to AOM/DSS. (**A**) Global comparison of genes differentially expressed in B6 control vs. AOM/DSS treated B6, and control *Irf1*^*−/−*^ vs. AOM/DSS-treated *Irf1*^*−/−*^, and calculated as fold change (log2). Genes differentially expressed specifically in *Irf1*^*−/−*^ (orange dots), specifically in B6 (purple) or common to both (green) are shown. (**B**) Heat map of genes significantly upregulated (FC ≥1.5, FDR ≤10^−2^) in both *Irf1*^*−/−*^ and B6 responses, or in *Irf1*^*−/−*^ only, or in B6 only. Enriched GO terms are shown to the side according to Benjamini-adjusted p-value significance. GSEA analysis of human IBD (**C**) and CRC (**D)** signatures as described in Materials and Methods.

Comparative analysis of the response of B6 and *Irf1*^*−/−*^ mice to AOM/DSS identified genes dysregulated (p < 10^−2^) in common in both strains as well as genes dysregulated only in B6 or only in *Irf1*^*−/−*^ mice (Fig. 3A). Analysis of GO terms for genes up-regulated in common included Inflammatory Response and Epithelial Development, including genes from the complement system (*C3, C4a, C4b*) and the TNF-activating receptor family (*Tnfrsf11b, Tnfrsf1b*). GO terms for genes exclusively upregulated in *Irf1*^*−/−*^ mutants included Inflammation and Leukocyte Activation, while B6 upregulated genes included Antigen Processing and Presentation and Innate Immune Response (Fig. 3B). These analyses suggest robust inflammatory response and tissue repair in both B6 and in *Irf1*^*−/−*^ mice; however, response of *Irf1*^*−/−*^ mice features a unique and much stronger implication of leukocytes, associated with a reduced signature of innate immune response. GSEA analysis was also performed, to compare the response of B6 and *Irf1*^*−/−*^ AOM/DSS-treated mice with the public GSE4183 dataset^37^ containing the expression profiles of human IBD and colorectal cancer tissues, and healthy controls (Fig. 3C). We observed an enrichment of the human IBD signature in both treated B6 and *Irf1*^*−/−*^ colon, but that response is quantitatively greater in *Irf1*^*−/−*^ mutants. This suggests stronger inflammation in the *Irf1*^*−/−*^ mutant with similarity to the pathological inflammatory state seen in human IBD (Fig. 3C). GSEA analysis also identified significant enrichment of a gene signature found upregulated in samples from human colorectal cancer tissue (compared to healthy tissues) uniquely in the colon of *Irf1*^*−/−*^ mutants treated with AOM/DSS (Fig. 3D). Importantly, the enrichment of this human CRC signature in the *Irf1*^*−/−*^ mice is found at a time point (D26) where tumors are not yet present. Therefore, after a single treatment of AOM and DSS, the *Irf1*^*−/−*^ mouse colons’ transcriptional profile is already enriched for genes upregulated in human IBD and human colorectal cancer.

### Irf1-dependent regulation of epithelial responses in the colon during CA-CRC

**Figure 4:**
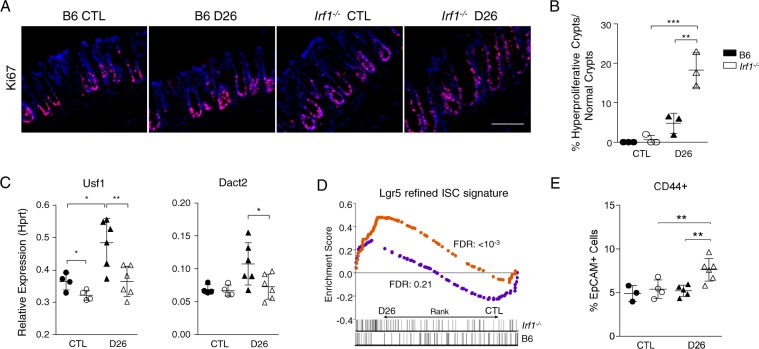
Epithelial dysregulation in colons of *Irf1*^*−/−*^ mutants after AOM/DSS treatment. (**A**) Representative images of immunofluorescence staining of frozen section for proliferation marker Ki67 (red), with control nuclear DNA stain DAPI (blue); Scale bars indicate 100μm. (**B**) Quantification of the number of hyperproliferative crypts compared to normal crypts from 5 fields of view examined under 10X magnification; Significance was determined using a one-way ANOVA with Bonferroni’s multiple-comparison test (**<0.01, ***<0.001). (**C**) Relative expression of negative regulators of cell cycle progression *Usf1* and *Dact2* in the colons of control and treated B6 (n = 4) and *Irf1*^*−/−*^ mutants (n = 6) measured by qRT-PCR and shown as relative expression compared to an internal *Hprt* control. (**D**) GSEA analysis of intestinal stem cell signatures as described in Materials and Methods. (**E**) Flow cytometry of intestinal epithelial cells in B6 and *Irf1*^*−/−*^ mice (n = 3 for B6 and *Irf1*^*−/−*^ CTL, n = 5 for B6 and *Irf1*^*−/−*^ D26) for CD44^+^ intestinal stem cells^,^ presented as percentage of total EpCAM^+^ epithelial cells.

We then investigated if the enriched human CRC signature in *Irf1*^*−/−*^ mice might be associated with early alterations in epithelial proliferation. Immunofluorescence analysis of cell proliferation marker Ki67 revealed an increase of “hyperproliferative epithelial crypts” in the colons of both B6 and *Irf1*^*−/−*^ mutants at D26, but in significantly greater abundance in *Irf1*^*−/−*^ mutants (Fig. 4A,B). Furthermore, the *Usf1* and *Dact2* genes are negative regulators of the cell cycle in response to Wnt signalling and as expected, these two genes are up-regulated in B6 colons but not in *Irf1*^*−/−*^ colons at D26 (Fig. 4C). These results suggest early transcriptional alterations in cell cycle regulation contributing to tumorigenesis in the *Irf1*^*−/−*^ colons in response to treatment. GSEA analysis detected enrichment of the highly proliferative *Lgr5*^+^ “intestinal stem cell (ISC) signature”^38^ in *Irf1*^*−/−*^ mice post-treatment (Fig. 4D). ISCs are major contributors to colorectal cancer development in response to sustained Wnt signalling^39^. FACS analysis also verified this signature, showing an increase in EpCAM^+^ CD44^+^ ISCs in the colons of *Irf1*^*−/−*^ mice at D26 (Fig. 4E). Globally, these results indicate that the *Irf1*^*−/−*^ mice respond to AOM/DSS treatment with an increase in epithelial proliferation compared to controls, as well as an increase in ISCs, and this, at a time (D26) preceding tumor emergence.

### Irf1-dependent regulation of cellular immune responses in the colon during CA-CRC

**Figure 5:**
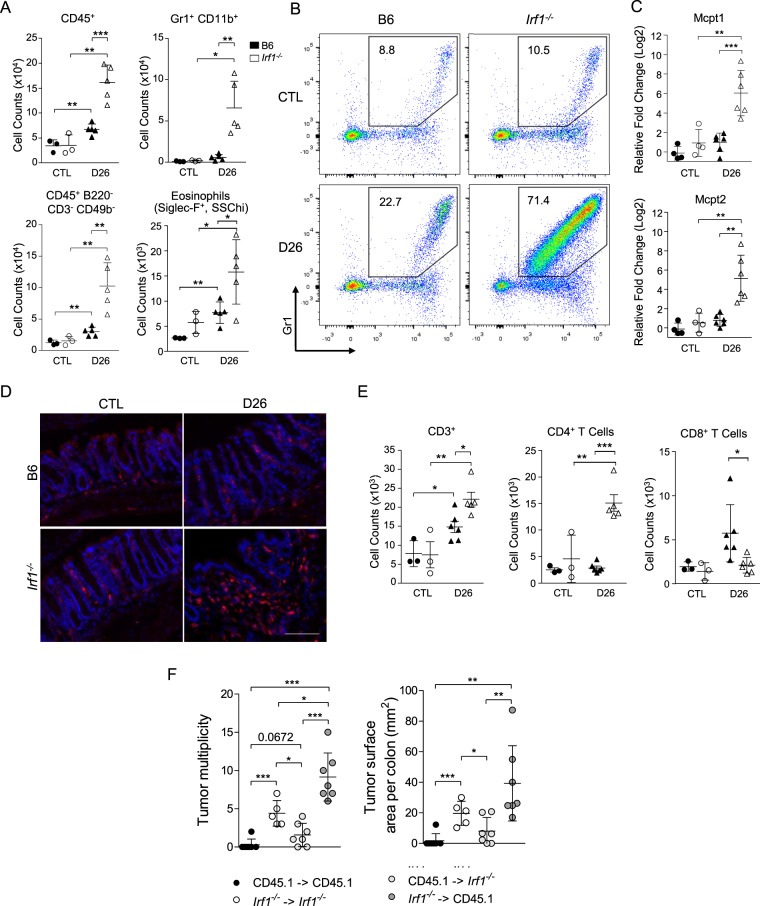
Elevated leukocytes infiltration in the colon of *Irf1*^*−/−*^ mice in response to AOM/DSS. Colon single cell suspensions from control and from treated (D26) B6 (filled symbols) and from *Irf1*^*−/−*^ mutants (empty symbols) were analyzed by flow cytometry. (**A**) Total counts of CD45^+^ cells, CD45^+^ B220/CD3/CD49b lineage negative cells, Gr1^+^ CD11b^+^ cells, and Siglec F^+^ cells are shown for individual mice along with means ± SD (n = 3 for CTL, n = 5 for B6 D26 and *Irf1*^*−/−*^ D26); Statistical significance was determined using unpaired Student’s t-tests (*<0.05, **<0.01, ***<0.001). (**B**) Representative FACS plots of Gr1^+^ CD11b^+^ cells. Numbers indicate mean % of myeloid cells using the gating window shown. (**C**) Relative expression of the mast cell markers *Mcpt1* and *Mcpt2* in the colon colons of control and treated B6 (n = 4) and *Irf1*^*−/−*^ mutants (n = 6) measured by qRT-PCR. (**D**) Representative images of Immunofluorescence staining for Cd11b^+^ myeloid (red) in the colon of control and treated mice, with control nuclear DNA stain DAPI (blue); Scale bars indicate 100 μm. (**E**) Total counts of CD3^+^, CD4^+^, and CD8^+^ T cells are shown for individual mice along with means ± SD (n = 3 for CTL, n = 5 for B6 D26 and *Irf1*^*−/−*^ D26); Statistical significance was determined using unpaired Student’s t-tests (* < 0.05, ** < 0.01, ***<0.001). (**F**) Susceptibility to CA-CRC in bone marrow chimeras (n = 7 for CD45.1 - >CD45.1, CD45.1 - > *Irf1*^*−/−*^, *Irf1*^*−/–*^ > CD45.1, n = 6 for *Irf1*^*–/*^ - > *Irf1*^*−/−*^) measured as tumor multiplicity and total tumor surface area. Mice were injected ip with AOM (7 mg/kg) once followed by three cycles of 2% (w/v) DSS exposure (4 days each) and sacrificed 6 weeks after the final DSS treatment. Statistical significance of inter-group differences was assessed using unpaired Student’s t-tests (*<0.05, **<0.01, ***<0.001).

Considering the impact of IRF1 inactivation on transcriptional responses in the colon, we conducted cellular immunophenotyping by flow cytometry of *Irf1*^*−/−*^ colons prior to and 26 days following treatment with AOM/DSS. While B6 controls showed a modest influx of CD45^+^ cells at D26, this influx was nearly three-fold greater in *Irf1*^*−/−*^ mutants (Fig. 5A). This infiltrate was composed mostly of myeloid cells (CD45^+^B220^−^CD3^−^CD49b^−^), primarily Gr1^+^CD11b^+^ neutrophils and inflammatory monocytes (Fig. 5A,B). *Irf1*^*−/−*^ mutants also showed an increase in eosinophils compared to controls (Fig. 5A), and RNA expression studies by qPCR also detected very significant expression of the mast cell-specific markers *Mcpt1* and *Mcpt2* in *Irf1*^*−/−*^ mutants compared to controls, suggesting that these cells are part of the myeloid infiltrate seen in *Irf1*^*−/−*^ colons (Fig. 5C). Overall, these results reveal that there is an accumulation of inflammatory granulocytes in *Irf1*^*−/−*^ at D26 compared to B6 mice, indicative of ongoing active inflammation in mutant colons. Finally, these findings were also validated by immunofluorescence analysis, showing Cd11b^+^ cells infiltrated into the lamina propria of *Irf1*^*−/−*^ colons at D26 (Fig. 5D). Increased infiltration of CD3^+^ T cells was also noted in treated *Irf1*^*−/−*^ mutants compared to B6 mice (Fig. 5E). This was predominantly a CD4^+^ T cell influx in *Irf1*^*−/−*^ mutants, corresponding with a more active state of inflammation in mutants. Importantly, there was also a unique increase in CD8^+^ T cell infiltration in B6 mice at D26 that was not seen in *Irf1*^*−/−*^ mutants. These results may indicate that cytotoxic responses by CD8^+^ T cells are important in anti-tumorigenic immune responses in B6 mice in response to treatment, and its absence in *Irf1*^*−/−*^ mutants may contribute to increased tumorigenesis.

Finally, to determine the overall contribution of this hematopoietic cells infiltrate to the CA-CRC susceptibility of *Irf1*^*−/−*^ mutants, we derived bone marrow (BM) chimeras and tested their susceptibility to AOM/DSS-induced CA-CRC susceptibility (Fig. 5F). These studies showed that transfer of *Irf1*^*−/−*^ mutant BM into irradiated B6 mice (CD45.1) confers significant CA-CRC susceptibility, at a level similar to that seen in *Irf1*^*−/−*^ mutant animals receiving *Irf1*^*−/−*^ mutant BM (Fig. 5F), whereas transfer of B6 (CD45.1) BM into irradiated *Irf1*^*−/−*^ mutant mice conveys significant protection against CA-CRC (Fig. 5F). These results confirm the critical role of hematopoietic cells in the hyper-sensitivity of *Irf1*^*−/−*^ mutants to CA-CRC. Altogether, these results suggest inadequate immune responses *in situ* in AOM/DSS-treated *Irf1*^*−/−*^ mutants that ultimately leads to compensatory leukocyte infiltration (predominantly inflammatory myeloid cells), possibly creating a tumor-promoting environment in these mice.

### IRF1 expression in human ulcerative colitis and colorectal cancer

**Figure 6:**
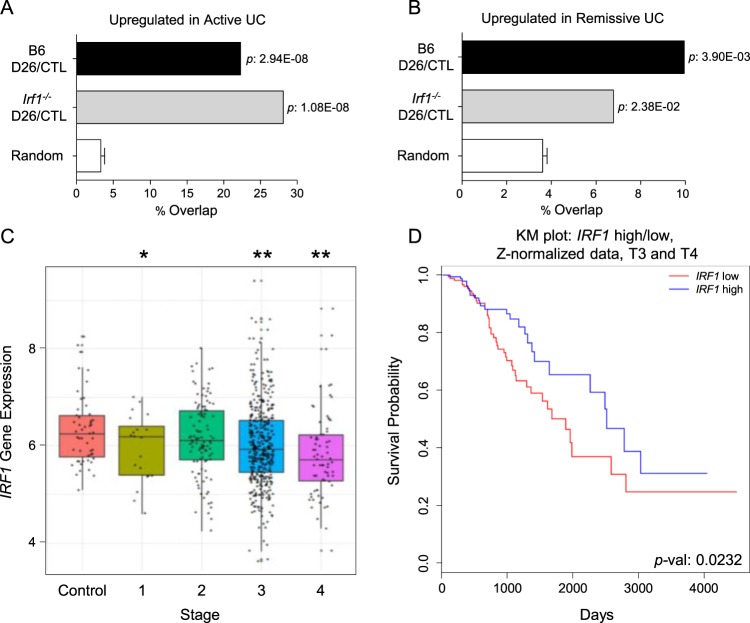
Association of Human *IRF1* with Ulcerative Colitis and Colorectal Cancer. Overlap between mouse RNAseq datasets for genes upregulated in response to AOM/DSS (at D26) for each genotype (B6, *Irf1*^*−/−*^; FC >1.5, FDR < 0.01) and genes upregulated in microarray datasets from active Human UC (**A**) or UC in remission (**B**) (FC >2, FDR <0.5). Fisher’s exact test was used to compute the significance of these overlaps compared to a randomly generated set of genes, with p-values indicated. (**C**) Analysis of TCGA database for *IRF1* mRNA expression in human CRC samples obtained at different stages of the disease. (**D**) Kaplan-Meier survival curve of Stage T3 and T4 patients with high or low *IRF1* gene expression level. Patients were classified by their relative expression of *IRF1* compared to the mean *IRF1* expression level of all Stage T3 and T4 CRC patients.

To assess a possible implication of *IRF1* in development of colonic inflammation and CA-CRC in humans, we first compared the AOM/DSS-induced transcriptional responses detected in the B6 and *Irf1*^*−/−*^ mutant colons to that of published colon transcript profiles associated with human UC^32^. The extent of overlap (percentage overlap, Fisher’s exact test *p* values) between genes upregulated in active UC and genes upregulated in the B6 and *Irf1*^*−/−*^ datasets indicated that the *Irf1*^*−/−*^ dataset had a greater and more significant overlap (28%) than the B6 datasets (22% overlap) (Fig. 6A). In comparison, the overlap between genes upregulated in UC patients undergoing remission was ~10% with the B6 dataset and 7% in *Irf1*^*−/−*^ (Fig. 6B), suggesting a stronger wound healing response in B6 controls. These results suggest a more severe and pathological inflammation in the colon of *Irf1*^*−/−*^ mutants than in B6 controls that more closely resembles transcriptional changes seen in human UC and that alterations in human *IRF1* expression may be associated with IBD in humans.

Furthermore, exploration of TCGA database for *IRF1* expression in human colorectal cancer patients revealed a statistically significant reduction of *IRF1* in tumors of Stages 1, 3, and 4 of colorectal cancer patients (Fig. 6C). Kaplan Meier survival curve analyses of T3 and T4 stage colorectal cancer tumors (Fig. 6D) with lower *IRF1* expression indicated a shorter survival of these patients, suggesting that decreased *IRF1* expression is a feature of human colorectal cancer progression.

Together, the observed CA-CRC hyper-susceptibility phenotype of *Irf1*^*−/−*^ mutant mice, the combined immunological and transcriptomic analysis of this phenotype, and the intersection noted with human IBD and human colorectal cancer datasets provide evidence for a strong role of *IRF1* in the development of pathological inflammation in IBD and its progression to colorectal cancer.

## Discussion

IRF1 plays a critical role in the ontogeny and function of the myeloid and lymphoid compartments of the immune system, in activation of intrinsic anti-microbial defenses and production of inflammatory cytokines activating early immune response by myeloid cells, resulting in primary defects in myeloid and lymphoid cell maturation and secondary defects in activation of T cell-specific immune responses upon its loss^40–43^. This causes susceptibility to bacterial and viral infections^44,45^. Likewise, IRF1 plays a critical role in activation of inflammatory response in mouse models of microbial^46^ or sterile inflammation^47^. Finally, human *IRF1* maps to the *IBD5* locus associated with susceptibility to IBD in GWAS^48^.

We therefore used *Irf1*^*−/−*^ mutant mice to study a possible cause to effect relationship between intestinal inflammation and susceptibility to colorectal cancer. We determined that *Irf1*^*−/−*^ mutants were extremely susceptible to CA-CRC, with greater tumor burden and mortality than A/J susceptible controls^19^. On the other hand, *Irf1*^*−/−*^ mutants were no more susceptible than B6 controls to AOM carcinogenesis alone, or to chronic exposure to DSS alone. These results differ from those published by Mannick *et al*.^16^ who observed that using a more aggressive DSS treatment protocol, *Irf1* mutant mice show increased inflammation and colonic crypt distortion compared to controls. So, while in our experimental model of CA-CRC, the milder DSS alone treatment did not reveal a differential colitic response in mutant mice, under prolonged conditions, *Irf1* mutant mice can show signs of increased susceptibility to DSS-induced colitis. Our work and that of Mannick *et al*.^16^ show that DSS alone cannot induce adenocarcinomas or at least not to the extent seen in our AOM/DSS model of CA-CRC. These results suggest that the combination of both carcinogenic and inflammatory insults were required for the cancer susceptibility phenotype of *Irf1*^*−/−*^ mutants.

Studies by RNA-seq identified differential transcriptional changes in B6 vs. *Irf1*^*−/−*^ colons following AOM/DSS revealing a much more robust and distinct inflammatory response in treated *Irf1*^*−/−*^ mutants (sustained infiltration of inflammatory leukocytes) compared to controls (more prominent antigen presentation). Likewise, FACS immunoprofiling of AOM/DSS-treated B6 and *Irf1*^*−/−*^ colons revealed a striking infiltration of myeloid cells (neutrophils, inflammatory monocytes) in *Irf1*^*−/−*^ mice compared to B6 mice. Importantly, studies in bone marrow chimeras established that hematopoietic cells are the main driver of CA-CRC development in *Irf1*^*−/−*^ mutants, as *Irf1*^*−/−*^ BM can transfer CA-CRC susceptibility to control B6, while B6 BM can protect *Irf1*^*−/−*^ mutants against CA-CRC.

Other prominent transcriptional responses were associated with the differentiation and maturation of epithelial cells that may reflect a more curative response in B6. GSEA analysis also identified a stronger intestinal stem cell signature in *Irf1*^*−/−*^ mutants, which may correspond to early signs of persistent proliferation of intestinal epithelial cells.

Together, these findings suggest the following mechanistic model for differential pathogenesis of CA-CRC and ultimate susceptibility of the *Irf1*^*−/−*^ mutant. In this model, dysregulation of Irf1 causes a defect in myeloid and lymphoid cell lineages. Such deficiencies in the number and function of DCs, CD8+ T cells, and NK cells have been described for *Irf1*^*−/−*^ mice^43^. Furthermore, our RNA-seq studies show that loss of Irf1 causes basal alterations in colonic immune and epithelial transcriptional programs (Fig. 2), which may promote a “tumor-permissive” microenvironment. Indeed, in this model, insult to the colonic mucosa during AOM/DSS treatment causes loss of epithelial integrity and exposure to luminal content of the intestine in both wildtype B6 and *Irf1*^*−/−*^ mice, but with distinct responses in each strain. In B6 mice, the initial response would involve local DC and macrophage-driven priming of a T cell-mediated pro-inflammatory response, whereas in *Irf1*^*−/−*^ mutants, deficiencies of the antigen-presenting cell compartment are concomitant to a compensatory response with massive infiltration of neutrophils, inflammatory monocytes, mast cells, and lymphoid cells.

Furthermore, disruption of intestinal barrier function can expose immune cells in the lamina propria to enteric bacteria, further enhancing neutrophil accumulation and inflammation. This is supported by RNA-seq data showing enrichment for antigen presentation uniquely in B6 mice, whereas *Irf1*^*−/−*^ mutants show enrichment for active leukocyte migration and inflammation. Though *Irf1*^*−/−*^ mice fail to display upregulation of antigen presentation programs, dendritic cell C-type lectin pattern recognition receptors (*Dectin-1, Dectin-2, Dap12)* and phagocytotic markers (*Sirpa, Cd68)* are increased, indicative of active pathogen detection. Furthermore, infiltration of pro-inflammatory cells in *Irf1*^*−/−*^ colons is readily detectable by FACS analysis (Fig. 5) and by RNA-seq (Fig. 3). Finally, the most highly differentially expressed genes post-treatment in *Irf1*^*−/−*^ mutants compared to B6 controls are mast cell proteases, *Mcpt1* and *Mcpt2* (Fig. 5C), which have been shown to recruit Gr1^+^ Cd11b^+^ cells in response to intestinal dysbiosis and promote their tumor-promoting capabilities in CA-CRC^49^. This sustained local leukocyte infiltration results in tissue damage and genotoxic conditions within the colonic microenvironment that increases susceptibility to AOM/DSS-induced carcinogenesis. Indeed, this is seen already at D26 by the sustained intestinal proliferation and increased intestinal stem cell populations in *Irf1*^*−/−*^ mice after treatment compared to controls (Fig. 4).

In humans, we propose that reduced expression or partial deficiency in IRF1 may predispose to IBD and subsequent progression to colorectal cancer in those individuals based on several lines of evidence. Firstly, human *IRF1* maps within the *IBD5* locus associated with susceptibility to IBD in humans^10^. We have recently developed a genome-wide annotation tool, the myeloid inflammation score (MIS) based on cistrome analysis of pro-inflammatory transcription factors and IFN-γ–induced transcriptomes in myeloid cells, to prioritize positional candidates at complex inflammatory disease loci^48,50,51^. Analysis of the 20 genes of the *IBD5* locus shows that *IRF1* has the highest score (MIS ~ 15). These, together with our observation that *Irf1* deficiency in untreated mice causes widespread transcriptional alterations of colonic immune and epithelial pathways provides compelling evidence that *IRF1* is the “morbid” gene at the IBD susceptibility locus *IBD5*.

Secondly, examination of the transcriptional response of *Irf1*^*−/−*^ mutants shows more extensive overlap with genes dysregulated in active UC lesions than that of B6 controls, suggesting that, following tissue injury, *Irf1* deficiency causes a pathological state in mice that resembles chronic inflammation seen in UC patients (Fig. 6B). Thirdly, GSEA analysis of transcriptional response in *Irf1*^*−/−*^ mutants identifies a highly statistically significant intersection with a human colorectal cancer transcript signature which is absent in B6 controls (Fig. 3D), and this, at an experimental time point where no tumors are present. This suggests that, following AOM/DSS-induced tissue injury, *Irf1* deficiency in the colon causes a pathological state in mice that resembles that seen in colorectal cancer patients. Finally, we note reduced *IRF1* mRNA expression in tumors from stage 3 and 4 colorectal cancer (Fig. 6C) that is associated with poorer prognosis and reduced survival times for these patients (Fig. 6D). Taken together, our combined studies in mouse models and publicly available human UC and colorectal cancer datasets suggest a critical role of IRF1 in inflammatory conditions of the colon, including progression to colorectal cancer.

## Supplementary information

Supplementary Information
