# Supplementary material for: Inactivation of Interferon Regulatory Factor 1 Causes Susceptibility to Colitis-Associated Colorectal Cancer
Source: Sci Rep. 2019 Dec 11;9:18897. doi: 10.1038/s41598-019-55378-2 (PMC6906452; doi:10.1038/s41598-019-55378-2)
Supplement: Supplementary file 1 — Supplementary Information [file 41598_2019_55378_MOESM1_ESM.pdf]

INACTIVATION OF INTERFERON REGULATORY FACTOR 1 CAUSES SUSCEPTIBILITY TO COLITIS-ASSOCIATED COLORECTAL CANCER

[1]Jeyakumar, Thiviya, [1]Fodil, Nassima, [1]Van Der Kraak, Lauren, [1]Meunier, Charles, [8]Cayrol, Romain, [6,7]McGregor, Kevin, [1,2]Langlais, David, [2,4,6,7]Greenwood, Celia M.T., [1,3,4,5,9]Beauchemin, Nicole and [1,2,3,9]Gros, Philippe

[1]Department of Biochemistry, McGill University, Montreal, QC, Canada
[2]Department of Human Genetics, McGill University, Montreal, QC, Canada
[3]Goodman Cancer Research Centre, McGill University, Montreal, QC, Canada
[4]Department of Oncology, McGill University, Montreal, QC, Canada
[5]Department of Medicine, McGill University, Montreal, QC, Canada
[6]Department of Epidemiology, Biostatistics and Occupational Health, McGill University, Montreal, QC, Canada
[7]Lady Davis Institute for Medical Research, Jewish General Hospital, Montreal, QC, Canada.
[8]Département de Pathologie et de Biologie Cellulaire, Université de Montréal, Montreal, QC, Canada.

[9]Equal contribution of the last two senior authors.

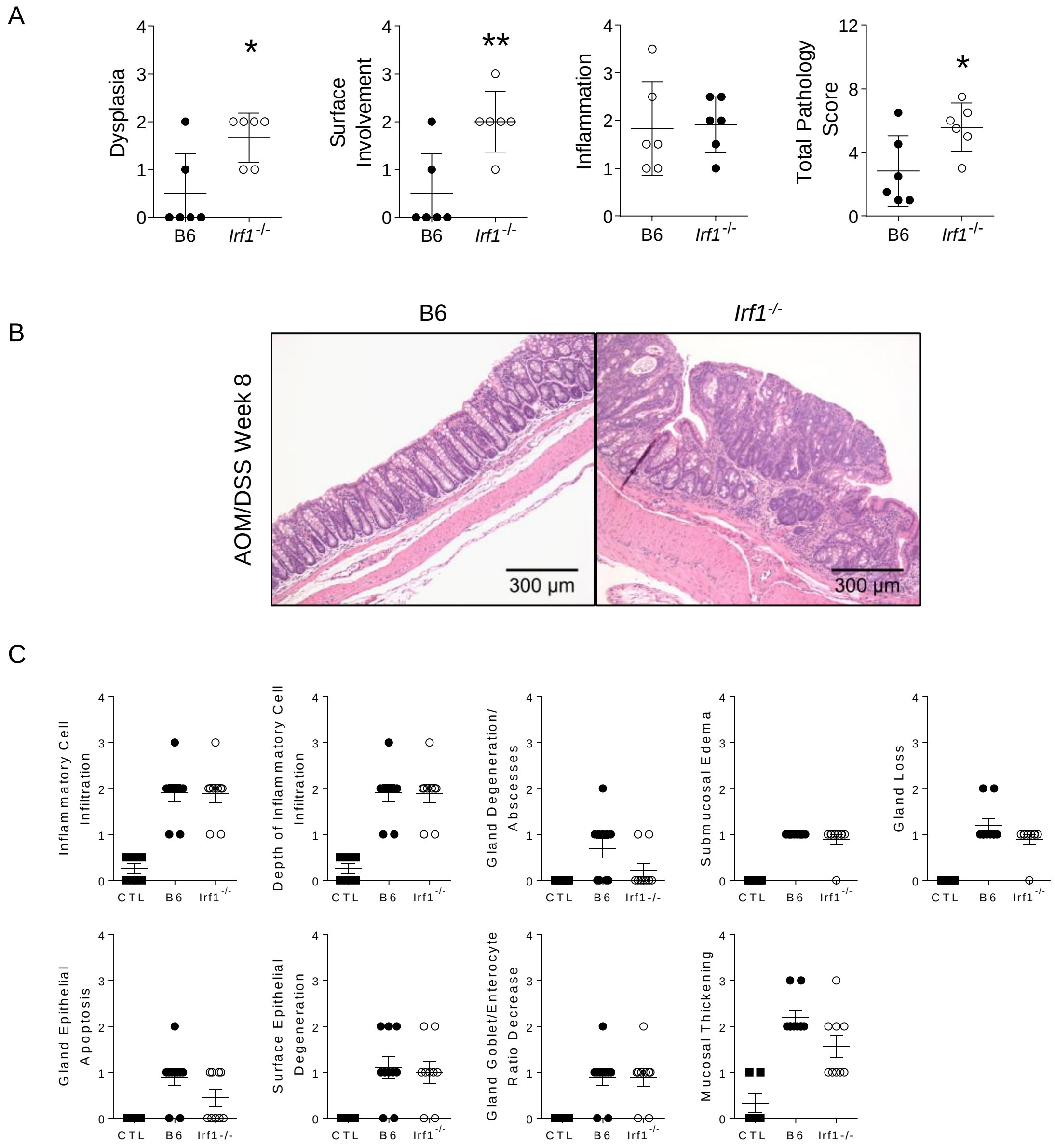

Supplemental Figure 1. Pathological changes in colons of *Irf1$^{-/-}$* mice after AOM/DSS treatment. Mice were injected i.p. with AOM (7 mg/kg) once followed by two cycles of 2% (w/v) DSS in drinking water (4 days each) and sacrificed 3 weeks after the final DSS treatment and colons were examined by histology. CA-CRC associated pathology was determined and quantified based on extent of dysplasia, colonic surface involvement and inflammation to establish a total pathological score (0-12) **(A)**, with representative H&E figures shown **(B)**. Chronic colitis was induced by treatment with three cycles of 2% (w/v) DSS in drinking water (4 days each). Mice were sacrificed 3 weeks after the final DSS treatment and colons were examined by histology. **(C)** Pathology scores (0-4) were determined for the following criteria: inflammatory cell infiltration, inflammatory cell depth, gland epithelial degeneration/abscesses, submucosal edema, gland loss, gland epithelial apoptosis, surface epithelial degeneration, gland goblet/enterocyte ratio decrease, and mucosal thickening, as described in Materials and Methods.

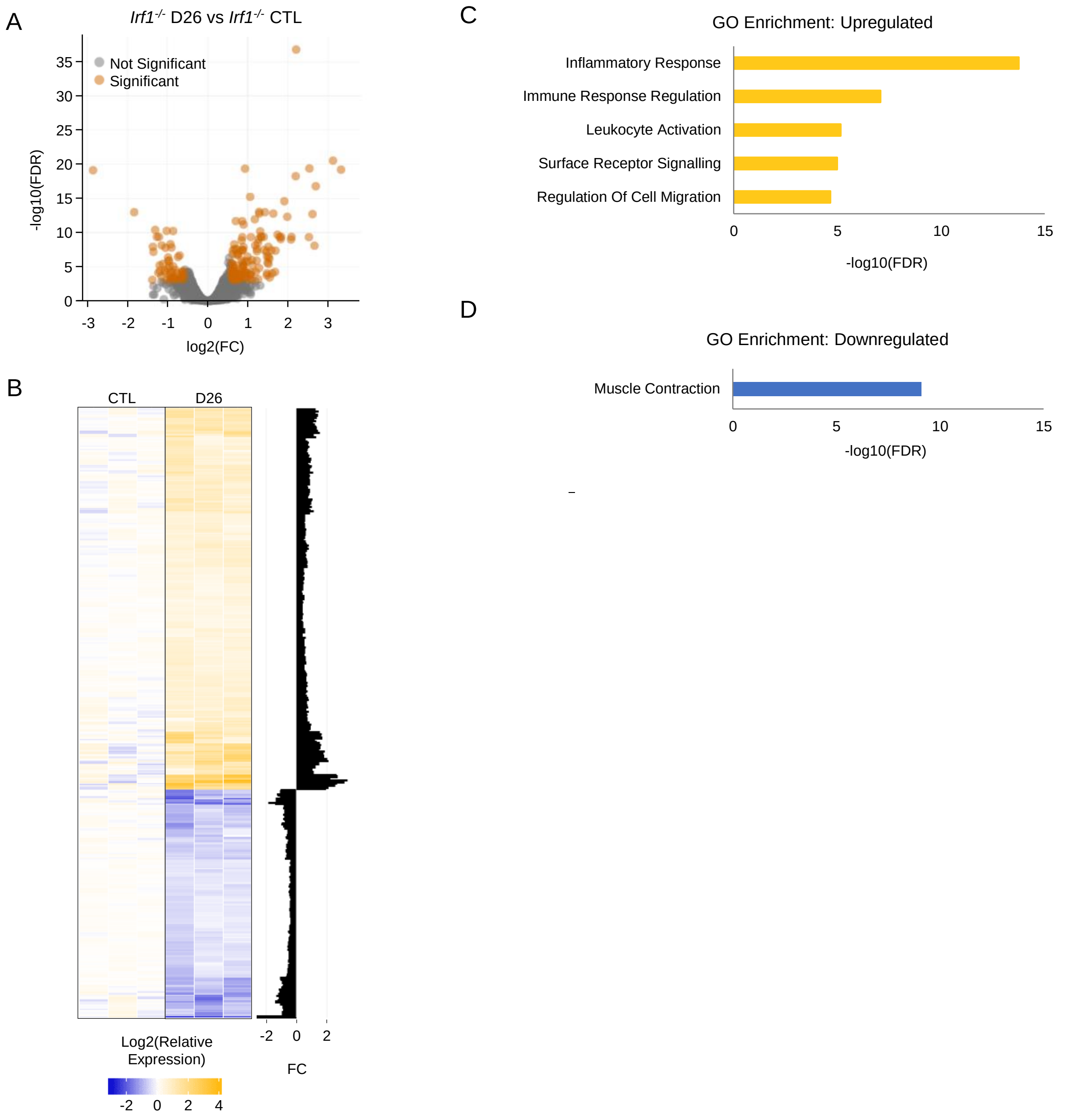

Supplemental Figure 2. Transcriptional response in the colon of *Irf1*$^{-/-}$ mice following combined treatment with AOM/DSS. RNA sequencing was performed on total RNA from the colons of control and treated (at day 26 post-treatment); Genes differentially expressed between control and treated *Irf1*$^{-/-}$ samples were identified, as described in legend to Fig 3. Significantly upregulated (FC ≥ 1.5, FDR ≤ 10^{-2}, n=188) and downregulated genes (FC ≤ -1.5, FDR ≤ 10^{-2}, n=76) are displayed by volcano plot (**A**) and heat map (**B**). Gene Ontology (GO) analysis for enrichment of specific GO terms in the group of upregulated (**C**) and downregulated (**D**) genes is shown.

Supplementary Table 1. Primers used for qPCR

Gene	Orientation	Sequence	Species
Dact2	sense	GCTGATGAGACCACTGTCCC	mouse
Dact2	antisense	GGACTCTTTCGAGATCACCTGTAG	mouse
Hprt (Ctl)	sense	TCAGTCAACGGGGGACATAAA	mouse
Hprt (Ctl)	antisense	GGGGCTGTACTGCTTAACCAG	mouse
Mcpt1	sense	GAGCTGGAGCTGAGGAGATTATTG	mouse
Mcpt1	antisense	TCCTCAGAACCTCTGTCCGT	mouse
Mcpt2	sense	ATAGGACAAGGAGATTCTGGGG	mouse
Mcpt2	antisense	TCCAGGGCAGGTAATAGGAG	mouse
Usf1	sense	CCACCCTTATTCCCCGAAGT	mouse
Usf1	antisense	GTTGTTGATCTTGTCCCGGC	mouse
